# Transcriptome analysis of *Aspergillus niger *grown on sugarcane bagasse

**DOI:** 10.1186/1754-6834-4-40

**Published:** 2011-10-18

**Authors:** Wagner R de Souza, Paula F de Gouvea, Marcela Savoldi, Iran Malavazi, Luciano A de Souza Bernardes, Maria Helena S Goldman, Ronald P de Vries, Juliana V de Castro Oliveira, Gustavo H Goldman

**Affiliations:** 1 Faculdade de Ciências Farmacêuticas de Ribeirão Preto, Universidade de São Paulo, Av do Café S/N, CEP 14040-903, Ribeirão Preto, São Paulo, Brazil; 2 Departamento de Genética e Evolução, Centro de Ciências Biológicas e da Saúde (CCBS), Universidade Federal de São Carlos, Brazil; 3 Departamento de Ciências Exatas e Tecnológicas, Universidade Estadual de Santa Cruz, Rodovia Ilhéus-Itabuna, km 16, CEP 45662-000, Ilhéus, Bahia, Brazil; 4 Faculdade de Filosofia, Ciências e Letras de Ribeirão Preto, Universidade de São Paulo, Avenida dos Bandeirantes, 3900, CEP 14040-901, Ribeirão Preto, São Paulo, Brazil; 5 CBS-KNAW Fungal Biodiversity Centre, Uppsalalaan 8, 3584 CT, Utrecht, The Netherlands; 6 Laboratório Nacional de Ciência e Tecnologia do Bioetanol (CTBE), Caixa Postal 6170, 13083-970 Campinas, São Paulo, Brazil

## Abstract

**Background:**

Considering that the costs of cellulases and hemicellulases contribute substantially to the price of bioethanol, new studies aimed at understanding and improving cellulase efficiency and productivity are of paramount importance. *Aspergillus niger *has been shown to produce a wide spectrum of polysaccharide hydrolytic enzymes. To understand how to improve enzymatic cocktails that can hydrolyze pretreated sugarcane bagasse, we used a genomics approach to investigate which genes and pathways are transcriptionally modulated during growth of *A. niger *on steam-exploded sugarcane bagasse (SEB).

**Results:**

Herein we report the main cellulase- and hemicellulase-encoding genes with increased expression during growth on SEB. We also sought to determine whether the mRNA accumulation of several SEB-induced genes encoding putative transporters is induced by xylose and dependent on glucose. We identified 18 (58% of *A. niger *predicted cellulases) and 21 (58% of *A. niger *predicted hemicellulases) cellulase- and hemicellulase-encoding genes, respectively, that were highly expressed during growth on SEB.

**Conclusions:**

Degradation of sugarcane bagasse requires production of many different enzymes which are regulated by the type and complexity of the available substrate. Our presently reported work opens new possibilities for understanding sugarcane biomass saccharification by *A. niger *hydrolases and for the construction of more efficient enzymatic cocktails for second-generation bioethanol.

## Background

Brazil is currently responsible for about 33% of the bioethanol produced worldwide and may play an important role in satisfying future bioethanol demand [1]. Nowadays there are more than 400 plants in operation crushing 625 million tons of sugarcane per year, with approximately one-half used for sugar and the other half used for bioethanol production [2]. In 2010, approximately 27.7 billion liters of bioethanol were produced on 8.0 million hectares of land [2]. The efficiency of sugarcane-to-ethanol production can still be increased through improvements in the agricultural and industrial phases of the production process [1,3,4]. Sugarcane bagasse (SB) contains one-third of the energy in sugarcane and is the source of all the energy needed in bioethanol mills. The other two-thirds are split between sucrose and the tops and leaves [4]. Presently the production of bioethanol in Brazil relies exclusively on first-generation technologies that are based on the utilization of the sucrose content of sugarcane. If sugarcane trash were collected and used for bioethanol production, it would generate an additional 3,700 to 4,000 L/ha bioethanol (9,700 to 10,000 L/ha total), thus reducing the land use requirement by 33% to 38%. The development of new and efficient technologies for hydrolysis of SB could improve this energetic balance considerably and create the basis for second-generation bioethanol. It is estimated that capital costs associated with lignocellulosic bioethanol are US$4.00/gallon and that these costs need to be reduced by more than half to be economically sustainable [5,6]. Complete substrate utilization is one of the prerequisites to rendering lignocellulosic ethanol processes economically competitive. This means that all types of sugars in lignocellulose must be converted to ethanol and that microorganisms must be obtained that efficiently perform this conversion under industrial conditions. Lignocellulose is composed of cellulose (40% to 50%), hemicellulose (25% to 35%) and lignin (15% to 20%) [7,8]. Glucose constitutes about 60% of the total sugars available in cellulosic biomaterial. Fermentation of the available sugars in cellulosic biomass presents a unique challenge because of the presence of other sugars, such as xylose and arabinose (C5 sugars). The degree of branching and the identity of the minor sugars in hemicelluloses tend to vary, depending upon the type of plant. The conversion of biomass to usable energy is not economical unless hemicellulose is used in addition to cellulose.

Considering that the costs of cellulases and hemicellulases contribute substantially to the price of bioethanol, new studies aimed at understanding and improving cellulase efficiency and productivity are of paramount importance. Filamentous fungi such as *Aspergillus niger *and *Hypocrea jecorina *(*Trichoderma reesei*) have been shown to produce a wide spectrum of polysaccharide hydrolytic enzymes. They are impressive producers of hydrolytic enzymes already applied in a variety of industrial processes, such as the food, feed, pulp, paper and textile industries. Recently, the genome sequences of *A. niger *and *T. reesei *have become available [9,10]. The *A. niger *and *T. reesei *genomes contain 14,600 and 9,129 genes, among which about 200 and 170, respectively, are involved in polysaccharide degradation. In *A. niger*, the expression of all major cellulases and hemicellulases is coregulated by the same inducer molecule (that is, D-xylose), but the induction mechanisms in *T. reesei *are more diverse. At least four different inductor molecules (that is, D-xylose, xylobiose, sophorose and lactose) have been described, but none of them has the potential to trigger the expression of all main cellulases and hemicellulases [11]. The xylanolytic/cellulolytic system in *A. niger *is regulated through the transcriptional activator XlnR. The *A. niger xlnR *transcription factor is a master regulator that activates enzymes of the xylanolytic system, a number of endocellulases and at least two cellobiohydrolases, but not that of β-glucosidase [11]. The major repressor protein regulating the carbon catabolite repression of genes involved in carbon metabolism in *Aspergillus *and *Trichoderma *is CreA [12,13]. Surprisingly, CreA is the only regulatory protein for which mediation of carbon repression has been demonstrated in fungi.

Although the source of cellulases is also other fungal species, enzymatic cocktails based on *T. reesei *dominate the market. However, a comparison of the genome sequences of *T. reseei *[10] and *A. niger *[9] shows that *A. niger *is more versatile in the range of cellulases, hemicellulases and esterases encoded, and the latter two groups of enzymes are likely to become more important if pretreatment steps become less extensively used. *A. niger *has become a very useful model fungus for basic studies in recent years because it has available annotated genome sequences, gene transfer systems and a variety of regulatory mutants. It is unlikely that commercial cellulases from *A. niger *will supplant those from *T. reesei*. The opportunity afforded by using the *A. niger *system is to provide new knowledge regarding the regulation of hydrolases (especially cellulases, hemicellulases and esterases) and accessory proteins that assist in the saccharification of complex substrates. To understand how enzymatic cocktails that can hydrolyze pretreated SBs can be improved, we used a genomics-based approach to investigate which genes and pathways are transcriptionally modulated during growth of *A. niger *on steam-exploded sugarcane bagasse (SEB). Herein we report the main cellulase- and hemicellulase-encoding genes that show increased expression during growth on SEB. We also sought to determine whether the mRNA accumulation of several SEB-induced genes encoding putative transporters is induced by xylose and repressed by glucose.

## Results

### Transcriptome analysis of *Aspergillus niger *grown on steam-exploded sugarcane bagasse

**Figure 1:**
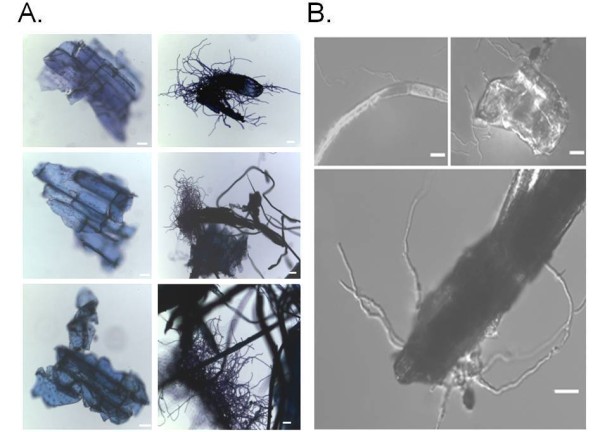
***Aspergillus niger *growth on SEB**. *A. niger *conidia were grown for 24 hours on batch cultivation medium without yeast extract supplemented with 0.5% steam-exploded sugarcane bagasse as carbon source. **(A) **Steam-exploded sugarcane bagasse (SEB) fragments stained with toluidine blue without any inoculation (left panel) and inoculated with *A. niger *and grown for 24 hours at 30°C (right panel). **(B) **Magnification of SEB inoculated with *A. niger *for 24 hours at 30°C. Bars = 20 μm.

**Figure 2:**
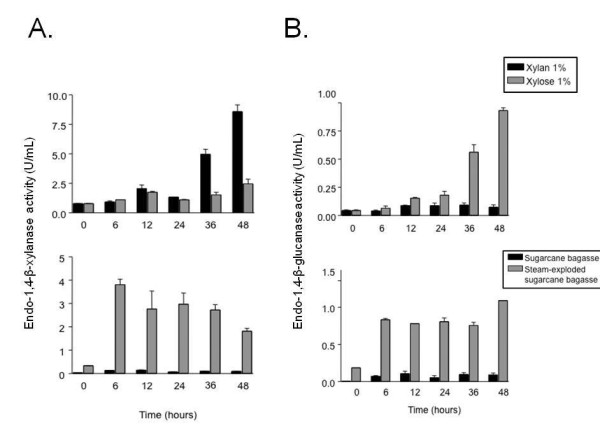
**Enzymatic activities of (A) endo-1,4-β-xylanase (xylanase) and (B) endo-1,4-β-glucanase (cellulase) in the presence of xylose, xylan and sugarcane bagasse (native or steam-exploded)**. One unit of enzyme activity is defined as the amount of enzyme required to release 1 μM D-xylose-reducing sugar equivalents per minute from arabinoxylan at pH 4.5 and 40°C. The results are the average of three repetitions, and the error bars represent standard deviations.

**Figure 3:**
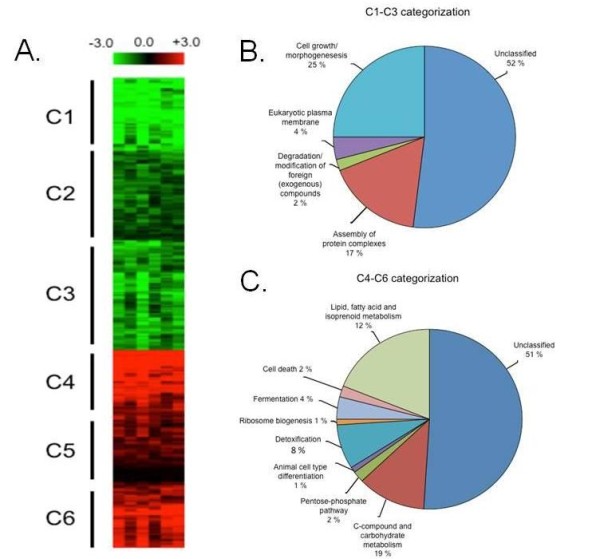
**Hierarchical clustering comparing the pattern of expression of *Aspergillus niger *grown on steam-exploded sugarcane bagasse (A)**. The color code displays the log_2 _ratio of cyanine 5 to cyanine 3 (Cy5/Cy3 ratio) for each time point with Cy3 used as the reference value (time point = 0, growth on fructose). The resulting significant data were visualized based on similar expression vectors using Euclidean distance and hierarchical clustering with an average linkage clustering method to view the whole data set. Clusters C1 to C3 and C4 to C6 show genes with decreased and increased mRNA accumulation, respectively, during growth of *A. niger*. Additional file 2, Table S2, and Additional file 3, Table S3, show genes that belong to these clusters. **(B) **and **(C) **The Functional Catalogue (FunCat) database http://mips.helmholtz-muenchen.de/proj/funcatDB/[14] functional annotation for the encoded proteins observed in clusters C1 to C6.

*A. niger *conidia are able to grow in liquid basic culture medium (BCM without yeast extract and supplemented with 0.5% wt/vol SEB as a carbon source (Figure 1). SEB fragments are not very regular and vary in size (Figure 1A, upper panels). After 24 hours of growth, gemlings are able to grow intimately into the SEB fragments, squeezing them (Figures 1A and 1B). As a first step toward verifying the induction of cellulase and hemicellulase production, we evaluated a time course of endo-1,4-β-xylanase (xylanase) and endo-1,4-β-glucanase (cellulase) activity when *A. niger *was grown in the presence of xylose, xylan, SB and SEB as carbon sources (Figure 2). Xylanase activity was higher in xylan than in any of the other three carbon sources, and it was almost absent in SB as single-carbon source (Figure 2A). Cellulase activity was higher in SEB than in any of the other three carbon sources (Figure 2B). Interestingly, cellulase activity was much higher in the presence of xylose than in the presence of xylan (Figure 2B, upper graph). As expected, the physical treatment of SB, that is, SEB, increased xylanase and cellulase activity dramatically (Figure 2, lower graphs). Curiously, during growth on SEB, xylanase and cellulase activity remained at about the same levels from 6 to 36 hours, but cellulose activity increased at 48 hours (Figure 2, lower graphs). We decided to choose 6, 12 and 24 hours as time points for our microarray analysis, with the aim of understanding early events involved in cellulases and xylanase induction. To gain insight into which hydrolytic enzyme-encoding genes and pathways are influenced by SEB, we determined the transcriptional profile of *A. niger*. We grew *A. niger *on 1% fructose (control) and transferred mycelia to 0.5% SEB as a single-carbon source for 6, 12 and 24 hours. In these experiments, our main aim was to focus on genes that have increased or decreased mRNA expression on SEB compared to the control. We were able to observe about 3,700 genes modulated at at least one time point (1,555 and 2,143 genes with log_2 _Cy5/Cy3 ratios ≥ 1 and ≤ 1, respectively) [GEO:GSE24798] http://www.ncbi.nlm.nih.gov/geo/query/acc.cgi?acc=GSE24798. The false discovery rate (FDR) was 1.18% (*P *= 1%; *P *= 0.01) (see Additional file 1, Table S1). These genes are involved in a variety of cellular processes, and they were classified based on Functional Catalogue (FunCat) database categories http://mips.helmholtz-muenchen.de/proj/funcatDB (Additional file 2, Table S2, and Additional file 3, Table S3, list the genes) [14]. Hierarchical clustering showed that these genes fall into six main clusters (Figure 3A). The first three clusters of genes (C1 to C3; *n *= 2,143 genes) (Figure 3A) showed the lowest expression levels on SEB. FunCat analysis [14] (Additional file 2, Table S2, and Additional file 3, Table S3) of the classified genes in these clusters showed a significant enrichment (*P *< 0.001) for (1) cell growth and morphogenesis, (2) assembly of protein complexes, (3) eukaryotic plasma membrane and (4) degradation of foreign exogenous compounds (Figure 3B). If the unclassified genes were not taken into account, the categories "cell growth and morphogenesis" and "assembly of protein complexes" would comprise 52% and 34% of these gene clusters, respectively (Figure 3B). In the "cell growth and morphogenesis" category, we observed genes involved in cell-cycle progression, such as a number of cyclin-encoding genes (An01g07040, An15g06500 and An07g08520) and cyclin-dependent kinases (An05g00280 and An09g04660). In addition, several genes encoding Ras-like proteins (An14g05530, An11g10320, An15g06650 and An5g00370) and signal transduction proteins involved in cell growth and monitoring nutrient conditions were expressed (G proteins such as An2g08000 and An02g01290 and protein kinases such as mitogen-activated protein kinase (An08g03240) and cAMP-dependent protein kinase catalytic subunit (An07g05060)). These results suggest that there is a reduction in growth progression during the 24-hour incubation with SEB.

The other three clusters of genes (C3 to C6; *n *= 1,555 genes) (Figure 3A) showed the highest expression levels on SEB. FunCat analysis [14] (Additional file 2, Table S2, and Additional file 3, Table S3) of the classified genes in these clusters showed enrichment of (1) C compound and carbohydrate metabolism; (2) lipid, fatty acid and isoprenoid metabolism; (3) cell death; (4) detoxification; (5) fermentation; (6) the pentose phosphate pathway; (7) animal cell-type differentiation; and (8) ribosome biogenesis (Figure 3C). Once more, if the unclassified genes were not taken into account, the categories "C compound and carbohydrate metabolism," "fermentation" and "pentose phosphate pathway" would comprise 50.36% of these gene clusters (Figure 3C). The categories "lipid, fatty acid and isoprenoid metabolism" and "detoxification" would comprise 25.18% and 15.28% of these gene clusters, respectively (Figure 3C). We first concentrated our attention on genes encoding cellulases and xylanases whose mRNA accumulation increased when *A. niger *was grown on SEB (Tables 1 and 2). The *A. niger *genome comprises 31 and 36 predicted cellulase- and hemicellulase-encoding genes, respectively [9]. We were able to observe increased expression of 18 (58%) and 21 (58%) cellulase- and hemicellulase-encoding genes, respectively, during growth on SEB (Tables 1 and 2). Interestingly, there is increased mRNA accumulation of several genes involved in fermentation, such as alcohol dehydrogenase (*AlcB*; An01g12170) and lactate dehydrogenase (*Ldh*; An11g09520), raising the possibility that lactate and alcohol fermentation occurs concomitantly with biomass saccharification. As expected, there is an intense modulation of several genes involved in C compound and carbohydrate metabolism and in the pentose phosphate pathway, which reflects the broad transport and metabolization of different classes of sugars during biomass utilization.

**Table 1:** Predicted *Aspergillus niger *cellulase-encoding genes that have increased mRNA accumulation during growth on steam-exploded sugarcane bagasse compared to growth on fructose reference control

Gene locus	Motifs*			GH family	SP	MS	Description	log_2 _Cy5/Cy3 ratio**		
						
	Sequence	Location	Orientation					6 hours	12 hours	24 hours
An03g03740				GH1	No	No	β-glucosidase (*bgl4*)	4.22	3.53	4.04
An11g02100	GGCTAG	249	1	GH1	Yes	No	β-glucosidase			
An15g04800	TTAGCC	-178	-1	GH3	Yes	No	β-1.2-D-glucosidase (*b2tom*)	1.90	0.37	1.85
An07g07630	GGCTAA	-180	1	GH3	Yes	No	β-glucosidase	4.88	3.67	3.21
An17g00520				GH3	No	No	β-glucosidase precursor (*bgluc*)	5.34	5.49	5.23
An18g03570				GH3	Yes	Yes	β-glucosidase (*bgl1*)	6.06	3.55	4.31
An11g00200				GH3	Yes	No	β-glucosidase (*bgln*)	6.23	5.34	5.94
An11g06090	CTAGCC GGCTAA	-69 -482	-1 1	GH3	Yes	No	β-glucosidase 2 (*bgl2*)	5.44	4.62	3.19
An03g01050				GH5	Yes	No	Endo-β-1.4-glucanase	3.75	0.92	2.58
An01g11670				GH5	Yes	No	Endo-β-1.4-glucanase A (*eglA*)	6.25	6.97	6.86
An07g08950	GGCTAA	-128	1	GH5	Yes	No	Endo-β-1.4-glucanase B	8.21	8.03	8.16
An08g01760	TTAGCC	-185	-1	GH6	Yes	No	Exocellobiohydrolase	5.07	3.47	2.73
An12g02220				GH6	Yes	Yes	Cellulose 1.4-β-cellobiosidase II (*cbh2*)	6.03	6.15	7.36
An07g09330				GH7	Yes	No	1.4-β-D-glucan cellobiohydrolase A precursor (*cbhA*)	5.95	5.83	3.89
An01g11660	GGCTAG TTAGCC	-417 -157	1 1	GH7	Yes	No	1.4-β-D-glucan cellobiohydrolase B precursor (*cbhB*)	7.43	6.91	6.58
An14g02760				GH12	Yes	No	Endoglucanase A (*eglA*)	7.88	6.85	6.35
An12g04610				GH61	Yes	No	Similarity to endoglucanase IV (*egl4*)	6.78	6.40	6.59
An01g01870	GGCTAA	-394	1	GH74	Yes	No	Avicelase III	4.69	4.52	4.15

GH = glycoside hydrolase; SP = signal peptide; MS = mass spectrometry; log_2 _Cy5/Cy3 ratio = base 2 logarithm ratio of cyanine 5 to cyanine 3. *Search of the promoter regions (500 bp upstream of putative translational start codon) of the displayed genes for the *A. niger *XlnR-binding sites containing either 5'-TTAGCC-3' or 5'-GGCTAG-3'. Location is relative to start codon. **log_2 _Cy5/Cy3 ratio observed in microarray hybridizations.

**Table 2:** Predicted *Aspergillus niger *hemicellulase-encoding genes that have increased mRNA accumulation during growth on steam-exploded sugarcane bagasse compared to growth on fructose reference control

	Motifs*							log_2 _Cy5/Cy3 ratio**		
						
Gene locus	Sequence	Location	Orientation	GH family	SP	MS	Description	6 hours	12 hours	24 hours
An12g01850				GH2	No		β-mannosidase	2.03	1.63	1.01
An01g09960	GGCTAA	-147 -133	1	GH3	Yes	No	Exo-1,4-β-xylosidase (*xlnD*)	6.70	5.85	5.51
An17g00300	TTAGCC	-487-9	-1	GH3	Yes	No	Bifunctional xylosidase-arabinosidase (*xarB*)	7.04	6.42	4.80
An03g00940	GGCTAA	-495 -288	1	GH10	Yes	Yes	Endo-1.4-β-xylanase A precursor (*xynA*)	6.77	5.86	5.52
An01g14600				GH11	Yes	No	Endo-1.4-β-xylanase	1.88	1.66	2.17
An15g04550	GGCTAA TTAGCC	-322 -270	1 -1	GH 11	Yes	No	Xylanase A (*xynA*)	6.21	6.21	6.19
An01g00780	GGCTAA	-216 124	1	GH11	Yes	Yes	Endo-1.4-β-xylanase B (*xynB*)	8.86	8.36	8.24
An01g03340	GGCTAA	-244	1	GH12	Yes	No	Xyloglucan-specific endo-β-1.4-glucanase	5.79	5.29	5.05
An14g01800				GH27	Yes	No	α-galactosidase D	3.01	1.63	1.23
An06g00170				GH27	Yes	No	α-galactosidase A	3.25	2.57	5.62
An02g11150				GH27	Yes	No	α-galactosidase (*aglB*)	4.16	2.00	1.38
An11g03120				GH43	No	No	Endo-1.4-β-xylanase (*xynD*)	2.09	1.70	1.95
An02g00140				GH43	No	No	Xylan-1.4-β-xylosidase (*xynB*)	2.88	1.43	1.34
An09g01190				GH43	Yes	No	Endo-1.5-α-arabinanase (*abnA*)	6.54	4.31	4.78
An08g01710				GH51	No	No	α-L-arabinofuranosidase (*abfA*)	5.69	2.66	4.38
An15g02300	CTAGCC TTAGCC	-39 -473 -421	-1 -1	GH54	Yes	No	Arabinofuranosidase B (*abfB*)	7.94	3.43	2.49
An03g00960	GGCTAA	-346	1	GH62	Yes	No	1.4-β-D-arabinoxylan arabinofuranohydrolase (*axhA*)	6.13	6.05	6.00
An14g05800	GGCTAG GGCTAA	-412 -277	1	GH67	Yes	No	α-glucuronidase (*aguA*)	9.12	8.36	7.72
An16g02760	GGCTAA	-245	1	GH95	No	No	α-fucosidase			
An12g02550	TTAGCC	-500	-1	CE1	Yes	No	Feruloyl esterase	7.18	7.24	7.87
An12g05010				CE1	Yes	No	Acetyl xylan esterase (*axeA*)	8.31	7.72	7.77

GH = glycoside hydrolase; SP = signal peptide; MS = mass spectrometry; log_2 _Cy5/Cy3 ratio = base 2 logarithm ratio of cyanine 5 to cyanine 3. *Results of a search of the promoter regions (500 bp upstream of the putative translational start codon) of the displayed genes for the *A. niger Xln*R binding sites containing either 5'-TTAGCC-3' or 5'-GGCTAG-3'. Location is relative to the start-codon. **log_2 _Cy5/Cy3 ratio observed in the microarray hybridizations. In the Orientation column, the numbers that were missing are the same.

### Validation of the microarray hybridization analysis

To validate some of our findings, we chose eight different genes from our microarray analysis whose mRNA shows increased accumulation when *A. niger *has grown on SEB. We designed Lux fluorescent probes and used real-time RT-PCR analysis to quantify their expression in *A. niger *grown on SEB for 6, 12 and 24 hours (Table 3). The measured quantity of a specific gene's mRNA in each of the treated samples was normalized using the comparative threshold (C_t_) values obtained for the actin mRNA amplifications run in the same plate. We also used β-tubulin as a normalizer and observed similar results (data not shown). The results were expressed as the number of times the genes showed increased expression when *A. niger *was grown on SEB compared to the control grown on fructose. We evaluated the mRNA accumulation of *xlnD *(An01g0960), encoding an exo-1,4-β-xylosidase; *xynB *(An01g00780), encoding an endoxylanase; *eglA *(An01g11670), encoding an endoglucanase; *eglB *(An07g08950), encoding an endoglucanase; *cbhA *(An07g09330), encoding a cellobiohydrolase; and *cbhB *(An01g11660), encoding a cellobiohydrolase. In addition, we quantified the mRNA accumulation of *xyrA *(An01g03780) and *xlnR *(An15g05810), which encode xylose reductase and the master transcription factor responsible for cellulase and xylanase induction in *A. niger*, respectively. Both genes showed greater expression during growth on SEB in our hybridization analysis (Additional file 3, Table S3). Interestingly, all genes tested showed increased mRNA accumulation at 6 hours, but then their mRNA accumulation decreased at 12 and 24 hours. However, all these genes showed, to different extents, increased mRNA accumulation when *A. niger *was grown on SEB compared to the noninducing conditions, that is, growth on fructose (Table 3). Thus it seems that our microarray hybridization approach is capable of providing information about *A. niger *gene expression modulation with a considerably high level of confidence.

**Table 3:** Gene expression measured by real-time RT-PCR of genes encoding *Aspergillus niger *cellulases, hemicellulases, xylose reductase and *xlnR *during growth on steam-exploded sugarcane bagasse

Gene and locus	Control (fructose)	SEB 6 hours	SEB 12 hours	SEB 24 hours
*cbhA *(An07g09330)	0.0086 ± 0.0008	0.3178 ± 0.0104	0.0477 ± 0.0013	0.0284 ± 0.0066
*cbhB *(An01g11660)	0.0023 ± 0.0000	4.2126 ± 0.1298	1.2376 ± 0.0629	1.6419 ± 0.3598
*eglA *(An01g11670)	0.0008 ± 0.0000	1.4762 ± 0.2621	0.7261 ± 0.0368	0.3397 ± 0.0740
*eglB *(An07g08950)	0.0062 ± 0.0004	3.7060 ± 0.0112	0.6213 ± 0.0248	0.1227 ± 0.0263
*xynB *(An01g00780)	0.0039 ± 0.0001	153.0746 ± 2.8399	9.8474 ± 0.6560	6.4386 ± 1.5611
*xlnD *(An01g09960)	0.0110 ± 0.0003	15.4472 ± 0.1495	0.7838 ± 0.0036	0.0617 ± 0.0149
*xlnR *(An15g05810)	0.76 ± 0.00	5.93 ± 0.62	1.71 ± 0.06	2.35 ± 0.24
*xyrA *(An01g03780)	0.33 ± 0.02	268.58 ± 6.22	45.84 ± 1.98	36.40 ± 1.11

SEB = steam-exploded sugarcane bagasse. The mRNA accumulation of several *A. niger *genes during growth on SEB. Cultures were grown for 24 hours at 30°C on batch cultivation medium (BCM) + 50 mM fructose, then the mycelial mass was transferred to BCM without yeast extract + steam-exploded sugarcane bagasse and grown for 6, 12 and 24 hours at 30°C. Real-time RT-PCR was used to quantify the mRNA. The measured quantity of a specific gene mRNA in each of the treated samples was normalized using the comparative threshold (C_t_) values obtained for the actin mRNA amplifications run in the same plate. The relative quantitation of specific gene and actin gene expression was determined by a standard curve (that is, C_t _values plotted against logarithm of DNA copy numbers). The results of four sets of experiments were combined for each determination. Data presented are means ± SD. The values represent the cDNA concentration of a specific gene divided by the actin cDNA concentration.

### Genes encoding putative transporters

We observed intense modulation of mRNA accumulation of several genes that encode predicted transporters potentially involved with sugar transport (see Additional file 3, Table S3). We concentrated our attention on seven of them that showed increased mRNA accumulation in the presence of SEB (Table 4). As a preliminary step toward assessing the function of some of these transporter-encoding genes, we performed real-time RT-PCR analysis. We validated the increased mRNA accumulation in the presence of SEB, and all of the genes showed increased mRNA accumulation to different extents when growth was induced by SEB (Table 5). Our laboratory is interested in identifying transporter-encoding genes that could be involved in xylose-transport. Thus we investigated whether the mRNA accumulation of these seven genes is modulated by xylose and affected by glucose (Table 6). All seven of these genes are induced by xylose and repressed by glucose to different extents (Table 6). Taken together, these results suggest that we were able to identify *A. niger *transporter-encoding genes that, based on their expression profiles, could be promising candidate xylose transporters.

**Table 4:** Predicted *Aspergillus niger *transporter-encoding genes that have increased mRNA accumulation during growth on steam-exploded sugarcane bagasse compared to growth on fructose reference control

Gene locus	Motifs*			Description	log_2 _Cy5/Cy3 ratio**		
			
	Sequence	Location	Orientation		6 hours	12 hours	24 hours
An01g00850	CTAGCC GGCTAA	-376 -385	-1 1	Similarity to xylose permease (*xylT*)	4.04	3.91	3.79
An06g00260	CTAGCC	-49	-1	Strong similarity to hexose transporter (*hxt5*)	5.87	4.36	6.23
An06g00620				Strong similarity to α-glucoside-hydrogen symporter (*mal11*)	7.19	4.73	5.96
An11g03700				Strong similarity to hexose transporter (*hxt1*)	1.33	1.97	2.64
An12g09270	GGCTAA	-356	1	Strong similarity to lactose permease (*lac12*)	3.52	4.44	2.94
An15g04270				Strong similarity to quinate transport protein (*qutD*)	5.07	6.02	8.29
An15g05440	TTAGCC	-6	-1	Strong similarity to high-affinity glucose transporter (*hgt1*)	3.85	3.02	2.86

log_2 _Cy5/Cy3 ratio = base 2 logarithm ratio of cyanine 5 to cyanine 3. *Results of a search of the promoter regions (500 bp upstream of putative translational start codon) of displayed genes for *A. niger Xln*R-binding sites containing either 5'-TTAGCC-3' or 5'-GGCTAG-3'. Location relative to start codon. **log_2 _Cy5/Cy3 ratio observed in microarray hybridizations.

**Table 5:** Gene expression measured by real-time RT-PCR of genes encoding *Aspergillus niger *putative transporters during growth on steam-exploded sugarcane bagasse

Gene locus	Control (fructose)	SEB 6 hours	SEB 12 hours	SEB 24 hours
An01g00850	0.064 ± 0.007	1.41 ± 0.01	1.38 ± 0.29	0.10 ± 0.02
An06g00260	0.21 ± 0.03	0.89 ± 0.09	1.70 ± 0.30	0.22 ± 0.02
An06g00620	2.25 ± 0.25	8.96 ± 0.93	8.87 ± 1.15	1.49 ± 0.19
An11g03700	0.396 ± 0.017	2.49 ± 0.08	3.02 ± 0.32	0.094 ± 0.019
An12g09270	0.96 ± 0.08	23.40 ± 3.50	4.28 ± 0.64	3.17 ± 0.61
An15g04270	0.036 ± 0.004	0.037 ± 0.006	0.13 ± 0.02	0.110 ± 0.009
An15g05440	0.021 ± 0.004	0.071 ± 0.002	0.041 ± 0.007	0.003 ± 0.001

SEB = steam-exploded sugarcane bagasse. The mRNA accumulation of several *A. niger *transporter-encoding genes during growth on SEB is shown. Cultures were grown for 24 hours at 30°C on batch cultivation medium (BCM) + 50 mM fructose, and the mycelia were transferred to BCM without yeast extract + SEB and grown for 6, 12, and 24 hours at 30°C. Real-time RT-PCR was used to quantify the mRNA. The measured quantity of a specific gene mRNA in each of the treated samples was normalized using the comparative threshold (C_t_) values obtained for the actin mRNA amplifications run in the same plate. The relative quantitation of a specific gene and actin gene expression was determined by drawing a standard curve (that is, C_t _values plotted against logarithm of DNA copy numbers). The results of four sets of experiments were combined for each determination. Data presented are means ± SD. The values represent the cDNA concentration of a specific gene divided by the actin cDNA concentration.

**Table 6:** Gene expression measured by real-time RT-PCR of genes encoding *Aspergillus niger *putative transporters in presence of xylose or xylose and glucose

Gene locus	Control (fructose)	Xylose 1 hour	Xylose 4 hours	Xylose + glucose 1 hour	Xylose + glucose 4 hours
An06g00620	0.08 ± 0.03	0.42 ± 0.07	0.38 ± 0.05	0.003 ± 0.00	0.03 ± 0.01
An15g04270	0.05 ± 0.00	0.04 ± 0.00	0.20 ± 0.00	0.04 ± 0.00	0.02 ± 0.00
An15g05440	0.34 ± 0.04	0.24 ± 0.03	0.56 ± 0.12	0.09 ± 0.05	0.19 ± 0.05
An11g03700	0.06 ± 0.00	0.04 ± 0.01	0.17 ± 0.01	0.009 ± 0.001	0.05 ± 0.01
An12g09270	0.29 ± 0.04	0.20 ± 0.02	0.56 ± 0.03	0.010 ± 0.001	0.20 ± 0.05
An01g00850	0.25 ± 0.01	0.16 ± 0.03	0.60 ± 0.02	0.13 ± 0.01	0.45 ± 0.17
An06g00260	0.70 ± 0.03	2.40 ± 0.22	2.30 ± 0.64	0.25 ± 0.01	0.36 ± 0.07

The mRNA accumulation of several *A. niger *transporter-encoding genes during growth on minimal medium (MM) + xylose or MM + xylose + glucose. Cultures from the *A. niger *N402 strain were grown for 24 hours at 30°C in batch cultivation medium (BCM) + 50 mM fructose and the mycelia were transferred to either BCM without yeast extract + 1% xylose or BCM without yeast extract + 1% xylose + 2% glucose and grown for one and four hours at 30°C. Real-time RT-PCR was used to quantify mRNA. The measured quantity of a specific gene mRNA in each of the treated samples was normalized using the comparative threshold (C_t_) values obtained for the actin mRNA amplifications run in the same plate. The relative quantitation of a specific gene and actin gene expression was determined by drawing a standard curve (that is, C_t _values plotted against logarithm of DNA copy numbers). The results of four sets of experiments were combined for each determination. Data presented are means ± SD. The values represent the cDNA concentration of a specific gene divided by the actin cDNA concentration.

### *In silico *identification of XlnR-binding motifs

The functional *A. niger *XlnR-binding sites identified contain either 5'-TTAGCC-3' or 5'-GGCTAG-3' [15-17]. A search of the promoter regions (500 bp upstream of the putative translational start codon) of the 1,516 genes with increased mRNA accumulation (Additional file 3, Table S3) revealed 461 genes with promoter regions containing at least one of these two binding sites and 63 genes that contain multiple binding sites (Additional file 4, Table S4). At least 15 putative transporter encoding genes, including An01g00850, An06g00260, An12g09770 and An15g05440, were tested for xylose induction (Additional file 4, Table S4, and Tables 4 through 6). The genes An06g00260, An12g09270, An01g00850 and An15g05440 were induced by xylose and repressed by glucose, suggesting that their putative XlnR-binding sites could be functional (see Table 6 and "Genes encoding putative transporters" section). Putative XlnR-binding sites were also found in the promoter regions of three genes encoding transcription factors (An05g00480 (*stuA*), encoding a transcription factor involved in differentiation; An01g08050 (*uaY*) a positive regulator of purine utilization; and An07g08880 (*cys*-3), a positive sulfur regulator). We observed XlnR-binding sites in the promoter regions of two genes encoding proteins that participate in the pentose catabolic and phosphate pathways (An01g03740 (D-xylose reductase) and An07g03160 (transaldolase), respectively). A search of the promoter regions of the genes encoding cellulases and hemicellulases (Tables 1 and 2) revealed that 8 (of a total of 18 cellulase-encoding genes) and 11 (of a total of 21 hemicellulase-encoding genes) promoters, respectively, contained at least one binding site, and that among those, 5 promoters had multiple binding sites.

### Secretome analysis of *Aspergillus niger *grown on steam-exploded sugarcane bagasse

To develop a preliminary notion of the proteins secreted by *A. niger *during growth on SB, we ran the proteins from the supernatant of a 24-hour culture on a polyacrylamide gel (data not shown). Nine bands of this gel were excised, and the proteins were eluted, digested with trypsin and identified by tandem mass spectrometry (MS-MS) (Table 7). Within these nine bands, we were able to identify seventeen different proteins that could be grouped into four different classes: (1) cellulases (*n *= 5), (2) xylanases and xylan hydrolysis-related enzymes (*n *= 7), (3) miscellaneous hydrolytic enzymes (*n *= 2) and (4) proteases (*n *= 3). Only three of the genes encoding these proteins were nonmodulated in our microarray analysis (glucan 1,4-α-glucosidase (*glaA*), An03g06550; tripeptidyl peptidase (unmamed gene), An14g02470; and acid α-amylase (*aamA*), An11g03340). All of the other proteins had corresponding positively modulated genes in our microarray analysis (see Additional file 3, Table S3).

**Table 7:** Protein identification by mass spectrometry from *Aspergillus niger *supernatants grown for 24 hours on steam-exploded sugarcane bagasse

Sample label	Protein name	Gene locus	NCBI GEO accession number	Theoretical molecular mass (kDa)	Score	Sequence coverage (%)	GE level (log_2 _Cy5/Cy3)
1	β-glucosidase	An18g03570	[GEO:215260053]	90	107	16	2.56 ± 0.03
1	Xylosidase (XlnD)	An01g09960	[GEO:145230215]	87	176	18	2.10 ± 0.05
2	Glucan 1,4-α-glucosidase (GlaA)	An03g06550	[GEO:145235763]	68	264	40	ND
2	Hypothetical protein	An14g02470	[GEO:145249068]	65	63	15	ND
3	1,4-β-D-glucan cellobiohydrolase B (CbhB)	An01g11660	[GEO:145235763]	56	264	40	6.59 ± 0.15
3	Acid α-amylase	An11g03340	[GEO:145243632]	55	63	13	ND
3	α-*N*-arabinofuranosidase B	An15g02300	[GEO:1168267]	52	256	23	7.94 ± 0.10
4	1,4-β-D-glucan cellobiohydrolase A	An07g09330	[GEO:74698499]	48	158	12	3.89 ± 0.20
4	Candidate cellulose 1,4-β-cellobiosidase II (Cbh2)	An12g02220	[GEO:145246118]	48	89	18	4.37 ± 0.05
5	Aspergillopepsin A	An14g04710	[GEO:1709632]	41	126	23	5.83 ± 0.09
6	Endo-1,4-β-xylanase A precursor	An03g00940	[GEO:145234695]	35	219	67	3.64 ± 0.06
6	1,4-β-D-arabinoxylan arabinofuranohydrolase (AxhA)	An03g00960	[GEO:145234699]	35	127	37	4.03 ± 0.01
6	Endo-1,4-β-xylanase C	An03g00940	[GEO:292495635]	35	230	57	3.64 ± 0.06
6	Protease B	An14g04710	[GEO:1585070]	34	136	38	5.83 ± 0.09
7	Chain A, crystal structure of ferulic acid esterase	An09g00120	[GEO:48425840]	28	105	40	6.17 ± 0.99
8	Endoglucanase A	An14g02760	[GEO:289595328]	25	99	22	4.36 ± 0.30
9	Xylanase	An01g00780	[GEO:13242071]	11	99	78	8.29 ± 0.04

NCBI GEO = National Center for Biotechnology Information Gene Expression Omnibus; kDa = kilodaltons; GE = gene expression; log_2 _Cy5/Cy3 ratio = base 2 logarithm ratio of cyanine 5 to cyanine 3; ND = no data. Data presented in right-hand column are means ± SD. Score column refers to Mowse scoring algorithm [45].

## Discussion

Sugarcane biomass residues are produced in great amounts in several countries. In Brazil, sugarcane production is remarkable because100% of bioethanol production as a biofuel is derived from sugarcane through sucrose fermentation by *Saccharomyces cerevisiae*. This process uses the sucrose accumulated in the sugarcane xylem and has conventionally been called "first-generation bioethanol." This ethanol corresponds to only one-third of the energy that can be extracted from sugarcane. The other two-thirds, derived from sugarcane tops, leaves and bagasse, are resistant to direct *S. cerevisiae *fermentation because this organism is not able to produce hydrolytic enzymes to degrade celluloses and hemicelluloses. In contrast, filamentous fungi have the ability to produce great amounts of hydrolytic enzymes. In this sense, enzymatic cocktails based on those enzymes have been proposed as an alternative method to break the resistance of those residues to fermentation. No detailed analysis has been published to date about the composition and structure of SB. However, it is quite likely that the composition and structure of celluloses (for example, the crystalline structure, number and extent of fibers) and hemicelluloses (for example, branching and different pentose concentrations) in SB are different from those of other biomass sources, such as wheat straw. Thus there is still a need to understand SB composition and structure and the enzymes necessary to completely hydrolyze it.

In our present study, we investigated the transcriptional response of *A. niger *strain N402 grown on pretreated SB. The genome annotation of this strain was used for the microarray slides. However, this strain is more closely related to the *A. niger *genomic DNA strain 3528.7 (ATCC 1015; American Type Culture Collection, Manassas, VA, USA) [GenBank:X52521], whose genome was recently published [18]. Unfortunately, the genome sequence of ATCC 1015 was not available when we started this project, thus we were not able to use it for the annotated genes for the microarray design. Our microarray analysis revealed about 3,700 genes differentially expressed in *A. niger *grown on SEB. The FunCat database functional annotation approach was used to determine whether distinct functional groups of genes were overrepresented within our data set. This analysis revealed "C compound and carbohydrate metabolism" as the most significantly enriched class (Additional file 2, Table S2). More than 50% of genes predicted in the genome of *A. niger *related to cellulases and xylanases were upregulated on SEB, and several of them were independently confirmed by real-time RT-PCR and mass spectrometry. There is considerable interest in the investigation of genes regulated at the transcriptional level during cellulosic biomass degradation by *A. niger *or *T. reesei *[19-22]. However, in all these previously published reports, the inducers were single-carbon sources and not complex substrates such as SB. An exception is an article reporting transcriptional profiling when *Neurospora crassa *was cultured on ground *Miscanthus gigantaeus *stems as the sole carbon source [23]. In comparing the data produced in our present work with the available expression data derived from *N. crassa *grown on *M. gigantaeus*, we observed that cellulases and hemicellulases present similar expression patterns, whereas the hydrolases specific to branching groups of the xylan backbone display less overlap in the compared systems. This difference could be attributed to the number of hydrolases, which vary widely among filamentous fungi. The *N. crassa *genome is predicted to be more limited in plant biomass degradation, with about 100 genes encoding hydrolases, whereas more than 170 could be identified in *A. niger *[9,10], suggesting that the latter fungus may utilize a wider variety of polysaccharide structures as its carbon source. Furthermore, a wide variety of glycosyl hydrolase (GH) families and member numbers are present in each GH family among fungi with genome sequences available [24,25]. Among the GH3 family, five members in the *N. crassa *genome and at least seventeen in the *A. niger *genome have been identified to date [9]. Only one member of the GH3 family has been detected in the *N. crassa *transcriptome (NCU00810), but our analysis revealed that eight members of the GH3 family are upregulated in *A. niger *grown on SEB (An15g04800, An07g07630, An17g00520, An18g03570, An11g00200 and An11g06090 (β-glucosidases), and An01g09960 and An017g00300 (xylosidases)). Moreover, the differential expression of hydrolases specific to xylan branching chains found in *A. niger *could be due to differences in the cell wall structures of sugarcane and *Miscanthus*. However, when we compared 149 *N. crassa *genes showing enrichment on *M. gigantaeus *(FunCat analysis of genes showing enrichment mainly for C compound and carbohydrate metabolism; see Support Information Dataset 1 published by Tian *et al*. [23]) with the whole *A. niger *SEB data set (Additional file 3, Table S3), we observed that 59% of *N. crassa *genes have corresponding *A. niger *homologues with mRNA accumulation also modulated on SEB (Additional file 5, Table S5). For example, genes encoding putative transporters that could be involved in carbohydrate transport, such as An1503940, An14g01600 and An12g07450 (*N. crassa *NCU04963, NCU05853 and NCU10021, respectively), have increased mRNA accumulation in the presence of both SEB and *Miscanthus *(Additional file 5, Table S5). Genes involved in the metabolism of pentoses, such as An01g10920 and An12g00030 (L-arabinitol 4-dehydrogenase (*N. crassa *NCU00643) and xylitol dehydrogenase (*N. crassa *NCU00891), respectively), also have increased mRNA accumulation in both substrates (Additional file 5, Table S5). These data strongly suggest that although diverse biomasses could have a specific impact on gene expression, some general features of these different residues affect mRNA accumulation in different filamentous fungi. We have also identified many genes whose expression level increased during growth on SEB and which encode proteins of unknown function that are conserved in other cellulolytic fungi (data not shown). Further deletion analysis of these genes may provide more information about their function.

D-xylose, the main component of xylan backbone, and other pentoses derived from xylan branching chains, such as arabinose, are assimilated by the pentose phosphate pathway. As proposed by Witteveen *et al*. [26], pentose catabolism in *A. niger *includes a reduction and oxidation step leading to D-xylulose, which is converted to D-xylulose-5-phosphate and enters the pentose phosphate pathway. We found a significant increase in mRNA accumulation of the genes encoding these enzymes (xylose reductase (An01g03740), xylitol dehydrogenase (An08g09380) and D-xylulokinase (An07g03140)) during growth of *A. niger *on SEB. These data are supported by promoter analysis of the genes with putative binding sites for XlnR (reported herein and by Foreman *et al*. [19]) and by the induction of their expression when *A. niger *grows on xylose [19]. Furthermore, the FunCat database classification "Pentose-phosphate pathway" (PPP) was represented in our analysis, suggesting that this complete catabolic pathway is upregulated when *A. niger *grows on SEB. Hasper *et al*. [27] showed that XlnR also regulates D-xylose reductase (*xyrA*), a gene from the pentose catabolic pathway, and it remains to be determined whether regulation at the mRNA level of other genes of the PPP is also XlnR-mediated.

Genes related to carbohydrate transport were also identified in the category "C compound and carbohydrate metabolism." The degradation of a complex biomass results in products such as glucose, cellobiose, xylobiose and xylose. Those products need to be transported into the fungal cytoplasm to be metabolized. Hence the upregulation of these genes should be required for better utilization of different sugars released from SB degradation. Our laboratory is interested in identifying transporter-encoding genes that could be involved in xylose transport. Toward this end we selected seven transporter-encoding genes and validated their expression levels by real-time RT-PCR. To check the specificity of these transporters, we tested genes with expression induced by xylose and repressed by glucose. We found that all of them were induced by SEB and showed increased mRNA accumulation when xylose was used as a single-carbon source. *S. cerevisiae *is a very poor xylose utilizer [28,29], and construction of strains able to transport xylose and/or other pentoses more efficiently by heterologous expression of these *A. niger *transporters may improve industrial fermentation of biomass hydrolytic products. None of these transporters or their homologues in other filamentous fungi have been characterized.

Nutrient sensing and signaling via pathways involving hexokinases, glucose transporter-like proteins, G protein-coupled receptors and protein kinases have been well-characterized in yeast [30-33]. Studies of multicellular filamentous fungi are at only a nascent stage. However, it is clear that the situation is considerably more complex in filamentous fungi than in yeast. This could be due to the preference of yeast for glucose fermentation instead of aerobic metabolism through the Krebs cycle as is the case in filamentous fungi. Although hydrolases, sugar transport and assimilation are essential for biomass degradation, we also observed other cellular responses induced when *A. niger *was exposed to SEB. We noticed decreased mRNA accumulation in several genes involved in cell growth and morphogenesis. Because *A. niger *grows an intermingled fashion with the set of SEB macroscopic fibers, it is not possible to accurately evaluate the dry weight of *A. niger *mycelia. We also tried to measure total protein concentration by Bradford and Biuret protein assays, but we were unable to obtain coherent results, most likely because of sugar interference (data not shown). However, it is possible that there is a slow biomass increase during the 24-hour exposure of *A. niger *mycelia to SEB. This slow growth is reflected in the reduced mRNA accumulation of several genes crucial to cell-cycle progression and signaling. It remains to be determined how significant these signal transduction pathways are for lignocellulosic sensing. Genes involved in fermentation have also shown increased mRNA accumulation during *A. niger *growth on SEB. There is evidence in the literature indicating that some fermentation occurs in addition to respiration through the Krebs cycle in filamentous fungi [34-36], and it is possible that, during growth on SEB, *A. niger *metabolizes sugars via fermentation pathways in addition to the pentose phosphate pathway and the Krebs cycle.

*A. niger xlnR *is a transcription factor that functions as a master regulator which activates genes that encode enzymes of the xylanolytic-cellulolytic enzyme system [11]. Electrophoretic mobility shift assays (EMSAs), DNase I footprinting, functional assays and comparisons of various xylanolytic promoters have helped to define the XlnR-binding consensus as 5'-GGCTAA-3' or 5'-GGCTAG-3' [14-16]. We were able to identify either one or both of these two motifs in several putative XlnR-binding sites in the promoters of the genes that were induced by SEB. Surprisingly, we were not able to identify them in a number of cellulase-, hemicellulase- and xylose-induced transporter genes, which suggests that there are other functional XlnR-binding motifs. Further characterization of the mRNA accumulation of these genes in the Δ*xlnR *mutant as well as DNA promoter binding assays (such as EMSA) will help to clarify whether these putative motifs are functional. Actually, the *A. oryzae *XlnR homologue binds to 5'-GGCTAA-3' and 5'-GGCTGA-3' [37,38] while *T. reesei *Xyr1 homologue binds to several 5'-GGC(A/T)3-3' motifs [39]. Therefore, it is possible that there are other XlnR-binding functional motifs different from those currently described. Further investigation is needed to clarify this issue.

## Conclusions

We have defined which genes are transcriptionally modulated in the early steps of growth on SEB. This study represents the first time that global transcriptional analysis has been performed for an industrial fungus grown on SB. Our analysis has revealed genes that are specifically induced when pretreated SB is used as a carbon source. Degradation of SB requires the production of many different enzymes which are regulated by the type and complexity of the available substrate. It is essential to understand which genes encoding hydrolytic enzymes are induced in the presence of SB if the intent is to produce enzymatic cocktails to hydrolyze this pretreated biomass. In addition to predicted cellulase genes, we identified genes encoding hemicellulases, carbohydrate esterases, β-glucosidases, β-xylosidases and other proteins predicted to have activity on carbohydrates in the *A. niger *transcriptome from SEB. In addition to these findings, we discovered that many genes with increased expression during growth on SEB encode proteins of unknown function and are conserved in other cellulolytic fungi. Some of these genes may encode putative proteins important for SEB saccharification, such as nonhydrolytic accessory proteins that increase or favor enzymatic efficiency. Our work opens new possibilities for the understanding of sugarcane biomass saccharification by *A. niger *hydrolases. The most important aspect of our study with regard to achieving these biotechnological goals is the comprehension of how gene expression is regulated during SEB saccharification. The heterologous expression of the genes that encode hydrolytic enzymes and their addition to enzymatic cocktails could improve enzymatic hydrolytic efficiency and consequently SEB saccharification. In addition, the construction of *A. niger *strains overexpressing some of the genes encoding these proteins can improve SEB saccharification. We observed a great number of transporter-encoding genes that may be functionally redundant or capable of transporting oligosaccharides produced by the action of secreted hydrolytic enzymes. Thus, by improving the efficiency of industrial fermentation of biomass hydrolytic products, it is possible to construct strains capable of transporting oligosaccharides by heterologous expression of *A. niger *transporters. This was recently achieved by expressing the *N. crassa *cellodextrin transport system in *S. cerevisiae *and promoting the efficient growth of this yeast strain on cellodextrins [40]. Further work involving several of the genes and pathways described herein will help to pave the way to more efficient enzymatic cocktails and second-generation bioethanol.

## Methods

### Strains and culture conditions

*A. niger *used was the N402 strain. The stock cultures were kept on silica beads with 7% milk (wt/vol) at 4°C. BCM (pH 5.5) was composed of 0.05% yeast extract (wt/vol), 50 ml/L salt solution (6 g/L NaNO_3_, 1.5 g/L KH_2_PO_4_, 0.5 g/L KCl and 0.5 g/L MgSO_4_), 200 μl/L trace elements (10 g/L ethylenediaminetetraacetic acid, 4.4 g/L ZnSO_4_·H_2_O, 1.0 g/L MnCl_2_·4H_2_O, 0.32 g/L CoCl_2_·6H_2_O, 0.315 g/L CuSO_4_·5H_2_O, 0.22 g/L (NH_4_)_6_Mo_7_O_24_·4H_2_O, 1.47 g/L CaCl_2_·2H_2_O and 1 g/L FeSO_4_·H_2_O) and a predetermined concentration of carbon source according to our experimental conditions. For cultivation in medium with SEB as the carbon source, the mycelia grown in the BCM were exhaustively washed with sterile distilled water and then transferred into BCM without 0.05% yeast extract but with 0.5% wt/vol of SEB as the carbon source. The BCM, BCM without yeast extract and BCM with SEB media contained (means ± SD): 3.1380 ± 0.1018 mg/ml, 0.0036 ± 0.004 mg/ml and 0.0073 ± 0.0012 mg/ml reducing sugars, respectively (data represent means of three experiments run in triplicate, with each medium type run three times using dinitrosalicylic acid (DNS)) [41]. SEB was kindly donated by Nardini Agroindustrial Ltda, Vista Alegre do Alto, São Paulo, Brazil, and was prepared as follows. *In natura *SB was treated with 14 kg/cm^2 ^water steam for eight minutes. The SEB was exhaustively washed with deionized water until reducing sugars were not detected by DNS [41]. After being washed, the SEB was kept completely dry at 40°C for several days and stored at room temperature. SEB fragments were stained with 0.05% toluidine blue for ten minutes and washed twice with water for five minutes.

### Xylan and steam-exploded sugarcane induction

*A. niger *spores were cultivated in complete medium (CM) at 30°C for three to five days and harvested by adding 20 ml of distilled water. The spore suspensions were inoculated to a final concentration of 1 × 10^6 ^spores per 30 ml of BCM culture. The spores were grown in BCM with 1% fructose (wt/vol) as the carbon source at 30°C for 24 hours and then transferred to either 1% xylose or 1% xylan (Sigma, St Louis, MO, USA) or 0.5% SEB (wt/vol) as the carbon source for 6, 12 or 24 hours. Mycelia were harvested by filtration through Whatman grade 1 filters (GE Healthcare, Grandview Blvd. Waukesha, WI, USA)), washed thoroughly with sterile water and quickly frozen in liquid nitrogen for further RNA extraction. The supernatant was kept at -20°C for enzymatic analysis.

### Determination of enzymatic activities

Xylanase (endo-1,4-β-xylanase) and cellulase (endo-1,4-β-glucanase) assays were performed using Azo-Xylan (Birchwood) and Azo-CM-Cellulose (both from Megazyme International, Bray, Ireland) as substrates, respectively, according to the manufacturer's protocols. Briefly, supernatant containing enzymes from SEB- or xylan-induced *A. niger *was mixed with 100 mM sodium acetate buffer (pH 4.5) in an appropriate volume. Reaction mixtures consisted of 0.5 ml of buffered enzyme preparation and 0.5 ml of substrate solution (1% wt/vol Azo-Xylan (Birchwood) for xylanase assay or 1% wt/vol Azo-CM-Cellulose for cellulase assay)). The samples were incubated at 40°C for ten minutes, and the reactions were interrupted by adding 2.5 ml of ethanol (95% vol/vol) with vigorous stirring. Nondegraded substrate precipitated by ethanol was removed by centrifugation at 1,000 × *g *for ten minutes, and the absorbance of the supernatant at 590 nm was measured. Enzymatic activity was determined using Mega-Calc™ software (Megazyme International). One unit of enzymatic activity was defined as the amount of enzyme required to release 1 mM D-xylose-reducing sugar equivalent per minute from arabinoxylan (pH 4.5) at 40°C.

### RNA extraction and real-time PCR reactions

After being harvested, mycelia were disrupted by grinding and total RNA was extracted using TRIzol reagent (Invitrogen/Life Technologies, Carlsbad, CA, USA). RNA (10 μg) from each treatment was fractionated in 2.2 M formaldehyde and 1.2% agarose gel, stained with ethidium bromide and visualized with UV light to check RNA integrity. The samples were then treated with RNAse-free DNAse as previously described [42], purified using the RNeasy Mini Kit (QIAGEN, Germantown, MD, USA)) and then quantified using a NanoDrop 2000 spectrophotometer (Thermo Fisher Scientific Inc., Waltham, MA, USA)).

All the PCR reactions were performed using the ABI 7500 Fast Real-Time PCR System (Applied Biosystems/Life Technologies, Carlsbad, CA, USA) and the TaqMan Universal PCR Master Mix kit (Applied Biosystems/Life Technologies). The reactions and calculations were performed according to the method described by Semighini *et al*. [42]. The primers, including the LUX™ fluorogenic primer (Invitrogen/Life Technologies), and probes used in this work are described in Additional file 6, Table S6, and the coefficients of the linear regression lines (standard curves) were added as described in Additional file 7, Table S7. The results of the microarray hybridizations were validated by real-time RT-PCR using an independent biological repetition different from those used for microarray hybridizations.

### Microarray slides construction and gene expression methods

To construct the microarray slides, we used the Agilent Technologies eArray software tool (https://earray.chem.agilent.com/earray/; Agilent Technologies, Inc, Santa Clara, CA, USA). Briefly, we uploaded 14,086 gene sequences representing the *A. niger *CBS513.88 strain gene sequences. This ORF number was carefully validated by comparing the sequences deposited in three databanks (CADRE (The Central Aspergillus Resource), JGI (Joint Genome Structure) and the BROAD Institute) with the aim of identifying and validating the sequences for probe design. Although some discrepancies in sequence length and number were detected, we were able to identify 14,086 ORFs, which were uploaded to eArray. On the basis of some quality parameter implemented in eArray (such as sequences with high scores for cross-hybridization potential throughout the genome and sequences for which no appropriate regions could be found as targets), 14,052 probes were designed from the uploaded sequence of CBS513.88. These probes were represented three times in the microarray slides, and the annotation based on the work of Pel *et al*. [9] was used to generate the annotation file used in the analysis. Therefore, the microarray slides comprised 45,220 features representing 1,417 eArray internal controls and 600 internal controls representing 60 randomly chosen ORFs (10 replicates).

To verify differential transcriptional activation of genes in *A. niger *grown on SEB for 6, 12 and 24 hours, we measured gene expression using microarray procedures. The gene expression analysis used in this work was carried out using custom-designed oligonucleotide slides (4 × 44 K microarray slides; Agilent Technologies, Inc), based on publicly available *A. niger *genome annotation. After RNA isolation and purification as described above, the samples were labeled with cyanine 3 (Cy3) or Cy5 dUTP using two-color microarray-based gene expression analysis (Quick Amp Labeling Kit; Agilent Technologies, Inc) according to the manufacturer's protocol. Initially, 5 μg of total RNA were incubated with Agilent Technologies RNA Spike-In Kit control probes (Spike A or B Mix). Prior to labeling, synthesis of cDNA was carried out by incubating the samples with 1.2 μl of T7 promoter primer and nuclease-free water in an appropriate volume. The template and primer were denatured by incubating the reaction at 65°C in a circulating water bath for ten minutes, and were placed on ice for five minutes after the reactions. We added Agilent Technologies cDNA Master Mix (4 μl of 5× First-Strand Buffer, 2 μl of 0.1 M dithiothreitol (DTT), 1 μl of 10 mM deoxyribonucleotide triphosphate mix, 1 μl of Moloney murine leukemia virus reverse transcriptase (MMLV-RT) and 0.5 μl of RNaseOut) to the samples, and the mixture was incubated at 40°C in a circulating water bath for two hours. Afterward the samples were moved to a 65°C circulating water bath and incubated for 15 minutes. cRNA amplification and labeling were performed by adding Agilent Technologies Transcription Master Mix (20 μl of 4× transcription buffer, 6 μl of 0.1 M DTT, 8 μl of nucleoside triphosphate mix, 6.4 μl of 50% PEG, 0.5 μl of RNaseOUT, 0.6 μl of inorganic pyrophosphatase, 0.8 μl of T7 RNA polymerase, 2.4 μl of Cy3-CTP added to control samples or Cy5-CTP added to treated samples, and 15.3 μl of nuclease-free water) to the samples and incubating the mixture in a circulating water bath at 40°C for two hours. The labeled cRNA was purified using the RNeasy Mini Kit and then quantified in the NanoDrop 2000 spectrophotometer.

For the hybridization, 825 ng of each labeled cRNA were mixed with Agilent Technologies fragmentation buffer (11 μl of 10× blocking agent, 2.2 μl of 25× fragmentation buffer and nuclease-free water to bring the volume to 52.8 μl) and incubated at 60°C for exactly 30 minutes to fragment RNA. The fragmentation was interrupted by adding 55 μl of 2× Hi-RPM GE Hybridization Buffer (Agilent Technologies, Inc). Next, 100 μl of sample were placed onto the microarray slide, which was mounted into the Agilent Microarray Hybridization Chamber. The hybridization was carried out in a microarray hybridization oven (G2545A; Agilent Technologies, Inc) set to 65°C for 17 hours. Afterward microarray slides were washed according to the manufacturer's instructions and scanned using a GenePix 4000B Microarray Scanner (Molecular Devices, Inc, Sunnyvale, CA, USA).

### Gene expression analysis

The extraction of data from TIFF image files generated by scanning microarray slides was performed using Feature Extraction version 9.5.3.1 software (Agilent Technologies, Inc) using the linear Lowess algorithm modeling method to obtain background subtracted and normalized intensity values. The dye-normalized values generated in the Feature Extraction software data files were used to upload the ExpressConverter version 2.1 file transformation tool (TM4 Microarray Software Suite; available at http://www.tm4.org/utilities.html), which conveniently converts the Agilent file format to MultiExperiment Viewer (MeV) file format, which is compatible with the TM4 software platform for microarray analysis. The MeV files were then uploaded into Microarray Data Analysis System (MIDAS) software (TM4 platform), where the resulting data were averaged from replicated genes on each array from two biological replicates of each treatment. The generated MeV files were analyzed using TIGR MeV software (TM4 platform, MultiExperiment Viewer (The Institute for Genomic Research, J. Craig Venter Institute, at http://jcvi.org)), where differentially expressed genes were statistically identified using a one-class *t*-test (*P *> 0.001). Significantly different genes were those whose mean log_2 _expression ratios for all included samples were statistically different from 0, which indicates the absence of gene modulation. The full data set was deposited in the National Center of Biotechnology Information Gene Expression Omnibus database [GEO:GSE24798] http://www.ncbi.nlm.nih.gov/geo/query/acc.cgi?acc=GSE24798.

To calculate the FDR, we used the method based on mixture distribution described by Allison *et al*. [43]. This method was implemented using GenStat statistical software package (VSN International Ltd, Hemel Hempstead, UK) with the *P *values as input, and FDRs (corrected *P *values) corresponding to each *P *value were generated. This test generates a large number of significance values, which under H_0 _(initial hypothesis) have a uniform distribution and under the alternative hypothesis can be modeled as a β- or truncated γ-density. The mixture distribution statistics estimate the parameters of the mixture distribution to derive the FDR. Further information can be obtained in the GenStat for Analysis of Microarray Data guide http://www.vsni.co.uk/downloads/genstat/release14/doc/MicroarrayGuide.pdf.

### Protein secretion analysis

The 30-ml supernatant from the sample induced with SEB for 24 hours was lyophilized and resuspended in 50 μl of distilled water. To separate secreted proteins, one-dimensional PAGE was performed using NuPAGE Novex Bis-Tris Mini Gels (Invitrogen/Life Technologies) according to the manufacturer's instructions. Briefly, 50 μl of sample were mixed with 4× NuPAGE LDS Sample Buffer, heated at 70°C for ten minutes and loaded onto the gel. The gel run was prepared 20× NuPAGE MES SDS Running Buffer to 950 ml of deionized water and performed at 125 mA with 1× SDS Running Buffer. To visualize the secreted proteins, the gel was stained with mass spectrometry-compatible Coomassie Brilliant Blue G-250 (USB Corp, Cleveland, OH, USA) and washed in 1% acetic acid before being imaged and processed for mass spectrometry.

### Matrix-assisted laser desorption/ionization time of flight mass spectrometry

Peptide mass fingerprinting of selected bands was carried out by in-gel trypsin digestion (Sequencing Grade Modified Trypsin; Promega, Madison, WI, USA) as reported previously [44]. Briefly, peptides were extracted from the gels using 60% acetonitrile in 0.2% trifluoroacetic acid (TFA), concentrated by vacuum drying and desalted using C18 reverse-phase microcolumns (OMIX Pipette Tips; Varian, Inc, Walnut Creek, CA, USA). Peptide elution from tip columns was performed directly into the mass spectrometer sample plate with 3 μl of matrix solution (α-cyano-4-hydroxycinnamic acid in 60% aqueous acetonitrile containing 0.2% TFA). Mass spectra were acquired using a 4800 Plus MALDI TOF/TOF Analyzer (AB SCIEX, Foster City, CA, USA) in reflector mode and externally calibrated using a mixture of peptide standards. Collision-induced dissociation MS-MS experiments of selected peptides were also performed. Proteins were identified by database searching with peptide *m/z *values using the Mascot sequence query program (Matrix Science, Inc, Boston, MA, USA) with the following search parameters: monoisotopic mass tolerance 0.05 Da, fragment mass tolerance 0.3 Da, methionine oxidation as a variable modification, and one missed tryptic cleavage allowed. Only Mowse algorithm [45] protein scores greater than 85 were considered statistically significant (*P *< 0.05)

## Abbreviations

bp: base pair; ha: hectare; kDa: kilodaltons; ORF: open reading frame; PEG: polyethylene glycol; RT-PCR: reverse transcriptase polymerase chain reaction.

## Competing interests

The authors declare that they have no competing interests.

## Authors' contributions

WRS, PFG, MS and IM performed most of the experiments. LASB performed the XlnR-binding site analysis. JCVO, MHSG and RPV performed data analysis and categorization. GHG wrote the manuscript; conceived, designed and coordinated this study; and is the principal investigator of this work. All authors read and approved the final manuscript.

## Supplementary Material

Additional file 1 **False discovery rate for the microarray data**. Table containing statistical analysis of the microarray data based on false discovery rate (FDR). The FDR was calculated by using the method based on "mixture distribution" described by Allison *et al*. [43]. This method was implemented using the GenStat statistical software package (VSN International Ltd, Hemel Hempstead, UK) with the *P *values as input, and FDRs (with corrected *P *values) corresponding to each *P *value were generated. Click here for file

Additional file 2 **Categorization of up- and downregulated genes of *Aspergillus niger *grown on steam-exploded sugarcane bagasse**. Tables showing differentially expressed (up- and downregulated) genes of *A. niger *grown on steam-exploded sugarcane bagasse (SEB) based on Functional Catalogue (FunCat) database categorization http://mips.helmholtz-muenchen.de/proj/funcatDB/. The genes were classified into different categories, which allowed us to observe the enrichment of these genes after growing *A. niger *on SEB. Click here for file

Additional file 3 **Time course of differentially expressed genes in *Aspergillus niger *grown on steam-exploded sugarcane bagasse**. Table showing gene description and log_2 _ratio of cyanine 5 to cyanine 3 (Cy5/Cy3 ratio) of differentially expressed genes (duplicates A and B) after time-course growth of *A. niger *on steam-exploded sugarcane bagasse (SEB). Click here for file

Additional file 4 ***In silico *identification of *Xln*R binding motifs**. Table presenting Gene ID, the predicted *A. niger Xln*R-binding sites of genes with increased mRNA accumulation, gene function and positions of the motifs. Click here for file

Additional file 5 **Comparison between genes showing increased expression in *Neurospora crassa *grown on *Miscanthus *and *Aspergillus niger *grown on steam-exploded sugarcane bagasse (C compounds and carbohydrate metabolism)**. Table showing upregulated genes for *N. crassa *(NCU) and *A. niger *(An) grown on *Miscanthus *and steam-exploded sugarcane bagasse (SEB), respectively, as well as gene function according to FunCat gene categorization. The *E*-value of the categorization is provided for *A. niger *genes. For *N. crassa E*-values, please refer to Support Information Dataset 1 published by Tian *et al*. [23]. Click here for file

Additional file 6 **Primers and probes used in this work**. Table containing the sequences and names of primers and probes used in this work. Click here for file

Additional file 7 **Coefficients of the linear regression lines (standard curves) obtained using Lux probes in this work**. Coefficients of the linear regression lines (standard curves) obtained by real-time RT-PCR analysis using Lux probes. Click here for file
