# Supplementary material for: Transcriptome analysis of Aspergillus niger grown on sugarcane bagasse
Source: Biotechnol Biofuels. 2011 Oct 18;4:40. doi: 10.1186/1754-6834-4-40 (PMC3219568; doi:10.1186/1754-6834-4-40)
Supplement: Additional file 7 — Coefficients of the linear regression lines (standard curves) obtained using Lux probes in this work. Coefficients of the linear regression lines (standard curves) obtained by real-time RT-PCR analysis using Lux probes. [file 1754-6834-4-40-S7.DOC]

**Additional file 7; Table S7 - Coefficients of the linear regression lines (standard curves) for the Lux probes used in this work.**

| **Gene ID (gene name)** | **Linear equation** | **Correlation coefficient (R^2^)** |
| --- | --- | --- |
| An08g03190 (*tubB*) | y = -3.40 + 41.12 | 0.9958 |
| An15g00560 (*actA*) | y = -3.49 + 45.78 | 0.9923 |
| An01g00850 (*xylT*) | y = -3.15 + 38.98 | 0.9954 |
| An02g11260 (*AmMst1*) | y = -3.40 + 41.62 | 0.9980 |
| An05g00730 (*hxt3*) | y = -3.33 + 40.42 | 0.9988 |
| An06g00260 (*hxt5*) | y = -3.34 + 40.79 | 0.9980 |
| An06g00620 (*mal11*) | y = -3.46 + 40.97 | 0.9813 |
| An11g03700 (*hxt1*) | y = -3.38 + 41.57 | 0.9965 |
| An12g09270 (*lac12*) | y = -3.54 + 42.28 | 0.9971 |
| An14g01600 (*hxtA*) | y = -3.31 + 40.37 | 0.9991 |
| An15g04270 (*qutD*) | y = -3.26 + 40.25 | 0.9988 |
| An15g05440 (*hgt1*) | y = -3.32 + 40.30 | 0.9975 |
| An01g03780 (*xyrA*) | y = -3.20 + 42.56 | 0.9992 |
| An01g00780 (*xynB*) | y = -3.11 + 41.99 | 0.9948 |
| An01g09960 (*xlnD*) | y = -3.02 + 42.30 | 0.9834 |
| An01g11660 (*cbhB*) | y = -3.17 + 43.41 | 0.9982 |
| An01g11670 (*eglA*) | y = -3.09 + 43.19 | 0.9906 |
| An07g08950 (*eglB*) | y = -3.25 + 43.48 | 0.9800 |
| An07g09330 (*cbhA*) | y = -3.22 + 43.59 | 0.9923 |
| An15g05810 (*xlnR*) | y = -3.06 + 42.79 | 0.9742 |
|  |  |  |
|  |  |  |
|  |  |  |
